# Cell Cycle, Division Rate, and Feeding of the Heterotroph *Phalacroma rotundatum* in a Chilean Fjord

**DOI:** 10.3390/microorganisms7100451

**Published:** 2019-10-14

**Authors:** Patricio A. Díaz, Iván Pérez-Santos, Gonzalo Álvarez, Michael Araya, Francisco Álvarez, Beatriz Reguera

**Affiliations:** 1 Centro i~mar, Universidad de Los Lagos, Casilla 557, Puerto Montt, Chile; ivan.perez@ulagos.cl; 2 CeBiB, Universidad de Los Lagos, Casilla 557, Puerto Montt, Chile; 3 Centro de Investigación Oceanográfica COPAS Sur-Austral, Universidad de Concepción, Campus Concepción, Concepción, Casilla 160-C, Chile; 4 Facultad de Ciencias del Mar, Departamento de Acuicultura, Universidad Católica del Norte, Larrondo 1281, Coquimbo, Casilla 117, Chile; gmalvarez@ucn.cl (G.Á.); falvarezsego@gmail.com (F.Á.); 5 Centro de Investigación y Desarrollo Tecnológico en Algas (CIDTA), Facultad de Ciencias del Mar, Larrondo 1281, Universidad Católica del Norte, Coquimbo Casilla 117, Chile; mmaraya@ucn.cl; 6 Instituto Español de Oceanografía (IEO), Centro Oceanográfico de Vigo, 36280 Vigo, Spain; beatriz.reguera@ieo.es

**Keywords:** *Phalacroma rotundatum*, cell cycle, in situ division rate, digestive vacuoles, small-scale physical processes, southern Chile

## Abstract

*Phalacroma rotundatum* is a rare cosmopolitan heterotrophic dinoflagellate. This species, included in the IOC-UNESCO Taxonomic Reference List of Harmful Microalgae, may be a diarrhetic shellfish poisoning (DSP) toxin vector, but little is known about its ecophysiology and behavior. A vertical net haul collected during the austral summer of 2018 in Reloncaví Sound (Chilean Patagonia) revealed an unusually abundant population of *P. rotundatum* and prompted intensive 24 h sampling on 16–17 January to study the cell cycle and feeding behavior of this species. Hydrographic measurements from a buoy revealed the local characteristic estuarine circulation, with a brackish surface layer (salinity 26–28) separated from saltier, colder bottom waters by a pycnocline at a depth modulated by the tidal regime. A high proportion of *P. rotundatum* cells were packed with digestive vacuoles (peak of 70% at 14:00), and phased cell division (*µ* = 0.46 d^−1^) occurred 3 h after sunrise. The division time (T_D_) was 2 h. This is the first cell cycle study of *P. rotundatum*. The results here disagree with those of previous field studies that considered asynchronous division in some *Dinophysis* species to be related to heterotrophic feeding. They also question the very specific prey requirements, *Tiarina fusus*, reported for *P. rotundatum* in northern Europe.

## 1. Introduction

*Phalacroma rotundatum* (Claparéde & Lachmann) Kofoid & Michener 1911 (=*Dinophysis rotundata*) is a rare, widely distributed, armored heterotrophic dinoflagellate. For many years, the relationship between *Dinophysis* and *Phalacroma* Stein—the latter long considered a synonym of *Dinophysis* [1,2]—has been a matter of controversy. Several authors [3,4] provided morphological and physiological reasons to support the original generic distinction between *Dinophysis* and *Phalacroma*. Species assigned to *Phalacroma* have narrow, horizontally projected cingular lists with an elevated epitheca visible in lateral view and are colorless, lacking the orange autofluorescence from cryptophycean-like chloroplasts, characteristic of phototrophic *Dinophysis* species [5]. Years later, molecular analysis of the large subunit of the ribosomal ribonucleic acid (LSU rDNA) of a large group of Dinophysiales supported the view that *Phalacroma* is a separate genus [6].

The feeding habits of *P. rotundatum* strains from the southern Kattegat, Denmark, were described by Hansen [7]. Observations from weekly seasonal samplings and from laboratory cultures revealed that the dinoflagellate fed upon a specific prey, the ciliate *Tiarina fusus,* using a feeding peduncle to perforate the ciliate’s lorica and suck its content into food vacuoles. This kind of phagotrophy, well-known in parasitic, mixotrophic, and heterotrophic dinoflagellates, was first described by Schnepf and Deichgräber [8] in *Paulsonella*, a parasite of the diatom *Streptotheca tamesis*.

Vegetative or asexual division in dinoflagellates of the order Dinophysiales (including *Dinophysis* and *Phalacroma* species) is by desmoschisis [9]. Each daughter cell inherits half of the maternal material and produces a new complementary half. After cytokinesis, the two daughter cells remain attached by their dorsal margins in an intercalary growth zone, the dorsal megacytic bridge, forming a pair of cells that remain together for a period of time that varies between species. Nevertheless, some parts of the maternal components are not evenly shared, as is the case with the large left sulcal lists (LSL) in *Dinophysis* species: one daughter cell inherits the posterior half of the left (large) sulcal list with ribs 2 and 3 (R2, R3), while the other daughter cell inherits the anterior half of the left sulcal list with the first rib (R1) [10]. These morphological details make it possible to recognize the paired cells (dividing) and the daughter cells with incomplete development of the left sulcal list (recently divided cells). Recognition of these forms has been used to estimate the in situ division rates of several species of *Dinophysis* using the mitotic and post-mitotic index approaches [10,11]. Sexual division in *Dinophysis* species includes the formation of small, gamete-like cells through a depauperating [12] division. Dimorphic cells are formed, with the newly generated half of the cell much smaller than the old half inherited from the mother cell [13,14]. The occurrence of these forms and their frequency were monitored for the first time in field populations of *Phalacroma* during the 24 h cycle study.

*P. rotundatum* is a widespread species in microplankton assemblages from North Atlantic coastal waters, but usually at very low (<10^2^ cells L^−1^) densities. Nevertheless, this rare species has drawn the attention of harmful algal bloom (HAB) experts ever since it was found to contain high levels of dinophysistoxin 1 (DTX-1). This toxin content was determined from a single sample of individually picked cells from Japan, analyzed by high-performance liquid chromatography (HPLC) with fluorescence detector, the most advanced method at that time [15]. As a result, *P. rotundatum* was included in the IOC-UNESCO Taxonomic Reference List of Harmful Microalgae [16]. Nevertheless, the ability of this species to produce toxins has been questioned. Toxin analyses of net samples rich in *P. rotundatum* collected during exceptional proliferation of this species were negative in the Gulf of Saint Lawrence, eastern Canada [17], and in the southern Adriatic Sea, Italy [17,18], or revealed toxin profiles identical with those of co-occurring *Dinophysis* species [17,18]. These results, together with observations of *P. rotundatum* digestive vacuoles with orange fluorescing pigments, led González-Gil et al. [19] to suggest that *P. rotundatum* is not a toxin producer, but a vector of DSP toxins taken up from tintinnid prey previously fed toxic *Dinophysis.* Still, the role of this species in the transfer of DSP toxins through the food web has been equated with that of toxin-producing *Dinophysis* species. Thus, it is important to know the environmental conditions which control its growth and behavior at aquaculture sites in Chile.

**Figure 1:**
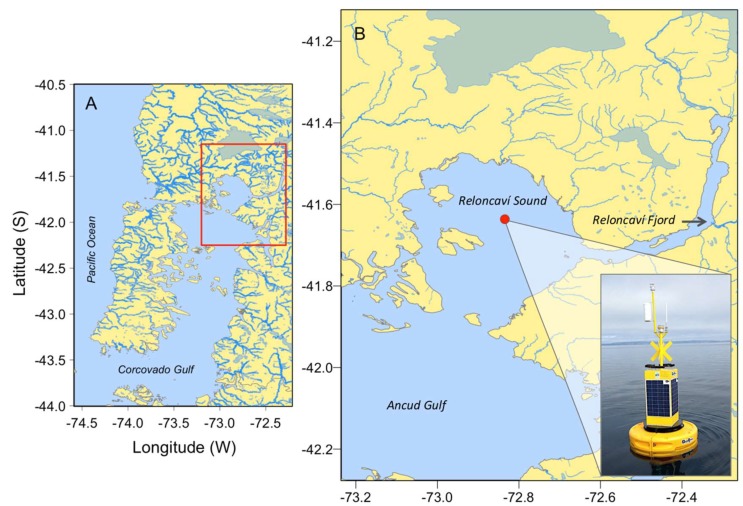
Map of the study area showing (**A**) northwestern Patagonia inland sea (the red box delimits Reloncaví Sound); (**B**) Reloncaví Sound in southern Chile. The position of the oceanographic buoy, where measurements were taken, is marked with a red circle.

During austral summer (16–17 January) 2018, unexpectedly high numbers of *P. rotundatum* were found in opportunistic vertical net hauls collected during routine maintenance visits to the institution oceanographic buoy deployed in Reloncaví Sound, southern Chile (Figure 1). This provided the opportunity to organize an ad hoc intensive 24 h sampling to gain information on the biology and behavior of this species.

This paper describes results from high-resolution physical and biological measurements carried out to describe environmental conditions associated with the proliferation of *P. rotundatum* in a Chilean Fjord. Description of the cell cycle and estimates of in situ division rate are given for the first time for this heterotrophic dinoflagellate. Ongoing molecular biology analyses may help to identify the ciliate prey, which seems to differ from the prey of *P. rotundatum* strains in northern Europe described by Hansen [7].

## 2. Material and Methods

### 2.1. Study Area

Reloncaví Sound is part of one of the largest fjord and channel systems in the world [20]. This system is characterized by its abrupt bathymetry, complex coastal morphology, and pronounced water column stratification determined by heavy seasonal runoff in spring–summer (snow melting), and rainfall in winter [21,22]. An intensive 24 h sampling was carried out during the austral summer from 16 to 17 January 2018, in Reloncaví Sound, northern Patagonia, at the same position as a buoy from the *i~mar* Oceanographic Centre (41°38.183′ S–72°50.069’ W) (Figure 1).

### 2.2. Meteorology, Sea Level, and Currents

Meteorological data, as well as sea level and current measurements, were obtained from the buoy. The meteorological station (Gill GMX500), a conductivity-temperature-depth (CTD AML model Metrec-XL) probe and an acoustic doppler current profiler (ADCP AWAC 400 kH) were deployed at a 3.5 m height, and 1.5 m and 8 m depth, respectively, and set up to collect data every hour. The CTD model Metrec-XL from AML Oceanographic instruments was also equipped with an optical sensor for dissolved oxygen (DO) and other sensors for turbidity, fluorescence, and pH. The ADCP, with a beam size of 2 m, was placed facing the seafloor.

### 2.3. Field Sampling

Vertical profiles of temperature and salinity, down to 240 m depth, were obtained every 3–4 h with a Seabird SBE 19 plus CTD profiler with a sampling rate of 8 Hz (8 measurements per second).

Vertical net (20 μm mesh) hauls from the surface to 20 m depth were collected hourly from 18:00 on 16 January to 18:00 on 17 January 2018 to observe morphological changes and estimate in situ division rate and frequency of recently fed (vacuolated) cells of the target specie. An aliquot (~50 mL) from each net sample was immediately fixed with acidic Lugol’s solution [23] and kept in a Falcon tube for cell counts.

### 2.4. Phalacroma Rotundatum Cell Cycle, Division Rate, and Vacuolation Estimates

Images were made of *Phalacroma* specimens undergoing cytokinesis, of recently fed vacuolated cells, and of life cycle stages, with a Micro-shot Microscope Camera (Guangzhou, China). To estimate the frequencies of cells undergoing different processes, net-haul (non-quantitative) samples were allowed to settle for 12 h using sedimentation columns of 10 mL. The whole surface of the sedimentation chamber was scanned at a magnification of ×100 with an Olympus CKX41 (Olympus, Tokyo, Japan) inverted microscope. In situ division rates were estimated, with a “post-mitotic index approach”, from the frequency of dividing (paired) and recently divided (incomplete development of the left sulcal list) cells, which were recognized by their distinct morphology as described in Reguera et al. [10], following the model of Carpenter and Chang [24]:

(1) $$\mu =\frac{1}{n\left(T_{c}+T_{r}\right)}\overset{n}{\underset{i=1}{\sum }}{\left(t_{s}\right)}_{i}\ln\left[1+f_{c}\left(t_{i}\right)+f_{r}\left(t_{i}\right)\right]$$

where *µ* is the daily average specific division rate, *fc*(*t_i_*) is the frequency of cells in the cytokinetic (or paired cells) phase (*c*), and *fr*(*t_i_*) is the half frequency of cells in the recently divided (incomplete development of the left sulcal list) (*r*) phase in the *i*th sample. *Tc* and *Tr* are the duration of the *c* and *r* phases, considered as “terminal events” sensu Carpenter and Chang [24] in this work; *n* is the number of samples taken in a 24 h cycle, and *t_s_* is the sampling interval in hours.

The duration of the selected terminal events, *T_c_* + *T_r_*, was estimated as the interval of time necessary for a cohort of cells to pass from one phase to the next; in this case, the time interval between the time *t*_0_—when the frequency of cells undergoing cytokinesis, *f_c_*, is maximum—and the time *t*_1_, when the fraction of recently divided cells, *f_r_*, is maximum:

(2) $$\frac{1}{2}\left(T_{c}+T_{r}\right)=\left(t_{0}-t_{1}\right)$$

where *T_c_*, *T_r_*, *t*_1_, and *t*_0_ are calculated after fitting a 5th degree Gaussian function to the frequency data.

The “maximum frequency approach” [25] was used to estimate *µ_min_*:

(3) $${µ}_{min}=Ln\;\left(1+f_{max}\right)$$

where *f_max_* is the maximal frequency of dividing plus recently divided cells, observed at the peak of division. This estimate is just a minimum estimate of the division rate and will approach the true value of *μ* only under specific conditions (i.e., very synchronized division, with the possibility of recognizing all the dividing or recently divided cells in a single sample).

To estimate the frequencies of cells containing food vacuoles, the ratio between vacuolated and the total number of specimen was calculated after examining a minimum of 100 cells (whenever possible) of *P. rotundatum* and expressing it as a percentage.

## 3. Results

### 3.1. Meteorological and Oceanographic Conditions and Microphytoplankton Community

**Figure 2:**
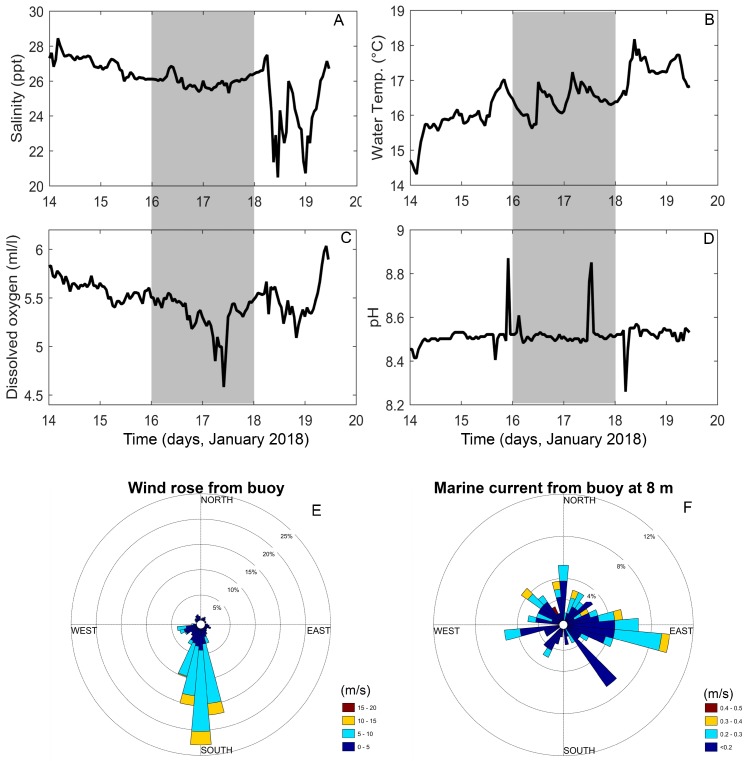
Oceanographic and meteorological variables from a buoy deployed in Reloncaví Sound during January 2018. (**A**) Salinity; (**B**) water temperature; (**C**) dissolved oxygen; (**D**) pH; (**E**) wind direction and velocity; (**F**) current direction and velocity. Shaded areas in figures A–D correspond to the sampling period.

Measurements from the oceanographic buoy showed an estuarine circulation during the sampling period (16–17 July 2018), with surface salinity ranging from 26 to 28 (Figure 2A). The surface temperature reflected the local summer patterns, with a conspicuous diurnal cycle (Figure 2B). Values of DO varied between 4.5 and 6.0 mL L^−1^, and pH from 8.4 to 8.8 (Figure 2C,D). Northerly surface currents were forced by the predominant southerly winds (Figure 2E), in addition to an easterly current due to freshwater inflow from Reloncaví Fjord and small-scale variability associated with the tidal regime (Figure 2F).

**Figure 3:**
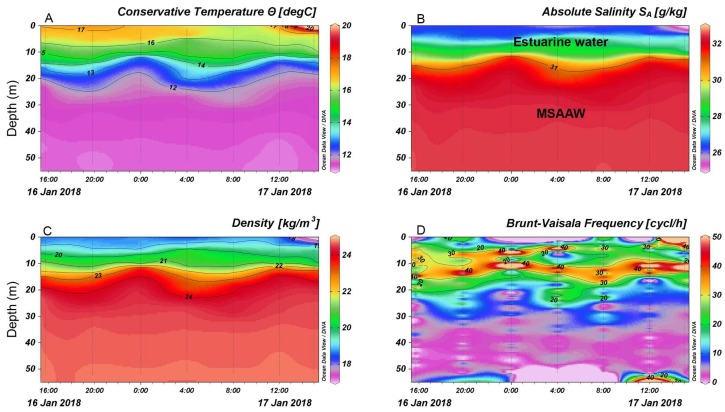
(**A**) Conservative temperature; (**B**) absolute salinity profiles, (**C**) water density; (**D**) Brunt–Vaisala frequency estimates at the oceanographic buoy station in Reloncaví Sound. Dotted lines correspond to CTD profiles.

The hydrographic profiler showed a two-layered water column structure: a warmer and fresher layer at the surface, and a colder and saltier layer below 20 m due to the inflow of Modified Subantarctic Water Mass (MSAAW), separated by a pycnocline at a depth controlled by the tidal regime (Figure 3A–D). These hydrographic conditions led to strong stratification at ~15 m depth, as revealed by the static stability values (Brunt–Väisälä frequency estimates) (Figure 3D), with a temperature gradient of 0.3–0.5 °C m^−1^.

Net-haul samples showed a microplankton community dominated by diatoms (*Rhizosolenia setigera*; *Corethron criophilum*; *Chaetoceros* spp.; *Pseudo-nitzschia delicatissima* complex; *Coscinodiscus* spp.) and a small contribution of dinoflagellates (*Lepidodinium chlorophorum*, *Dinophysis acuminata*, *Gyrodinium* spp.; *Protoperidinium* spp.), of which *P. rotundatum* was the most abundant species.

### 3.2. Cell Cycle, In Situ Division Rates, and Feeding Status of P. rotundtum

**Figure 4:**
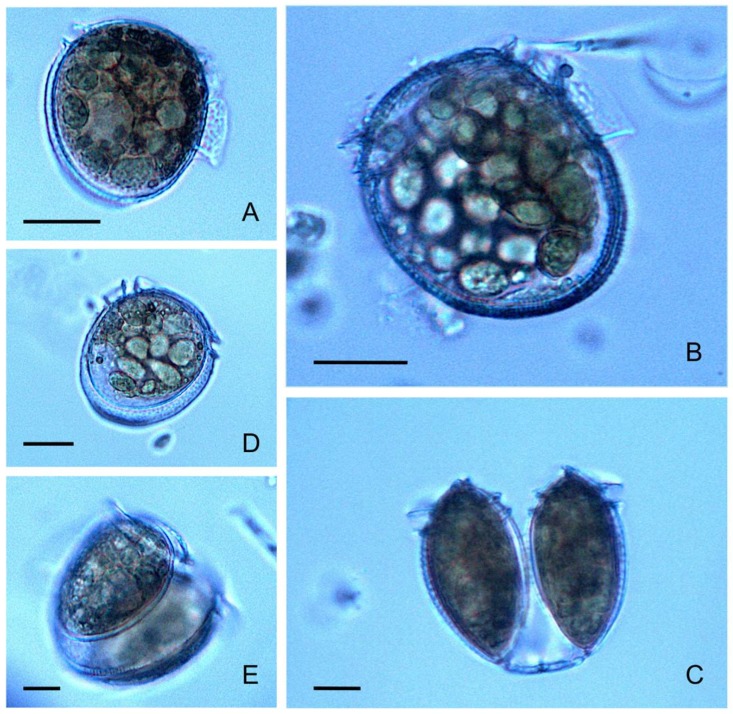
*Phalacroma rotundatum* micrographs showing different cell cycle (vegetative) phases and life cycle stages. (**A**,**B**) Fully developed single cells showing abundant digestive vacuoles; (**C**) recently divided vegetative paired cells showing the megacytic bridge; (**D**) recently divided intermediate-sized cell of *P. rotundatum,* with digestive vacuoles and incomplete development of the left sulcal list; (**E**) dimorphic paired cells undergoing depauperating division; scale bar = 20 μm applies to all frames.

*P. rotundatum* cells collected with vertical hauls appeared healthy, with many cells showing signs (digestive vacuoles) of recent feeding (Figure 4A,B) and undergoing cell division (Figure 4C,D). Morphological variability provided evidence of different phases of the cell cycle, such as cytokinesis (paired cells) (Figure 4C), recently divided cells (incomplete development of the left sulcal list) (Figure 4D), and different nutritional status (well-fed, vacuolated, or starved), making it possible to estimate the division rates and explore the existence of circadian rhythms.

**Figure 5:**
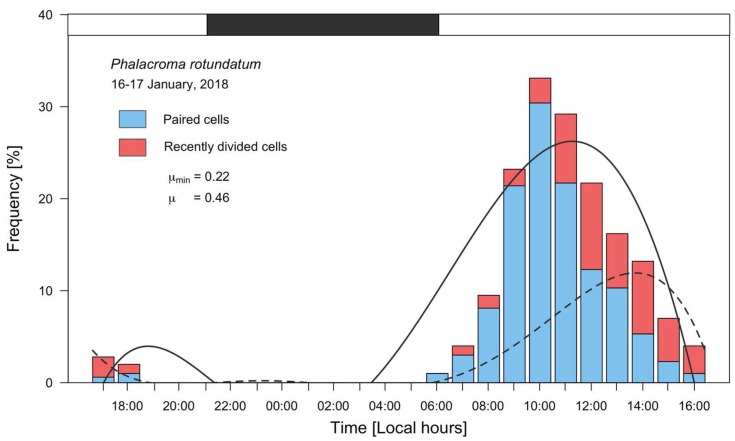
Distribution of frequencies of vegetative paired (blue bars) and recently divided cells (red bars) of *P. rotundatum* from 16 to 17 January 2018, fitted to a 5^th^ degree polynomial curve. The solid line curve corresponds to paired cells, and the dashed line to recently divided cells; the black segment in top bar indicates the dark (sunset to sunrise) period.

Frequency distributions of dividing (*f_c_*) and recently divided (*f_r_*) cells of *P. rotundatum* showed a clear-cut, phased cell division during this study (Figure 5), revealing the same cell cycle pattern as already described for *Dinophysis* species [10]. The maximum frequency of paired cells (30.4%*)* was observed at 10:00, 3 h after sunrise, and the maximum frequency of recently divided cells (9.4%) at 12:00. Thus, the time lag between the peaks of cytokinesis and sulcal list regeneration was 2 h. Estimates of *µ* and *µ_min_* were 0.46 d^−1^ and 0.22 d^−1^, respectively.

**Figure 6:**
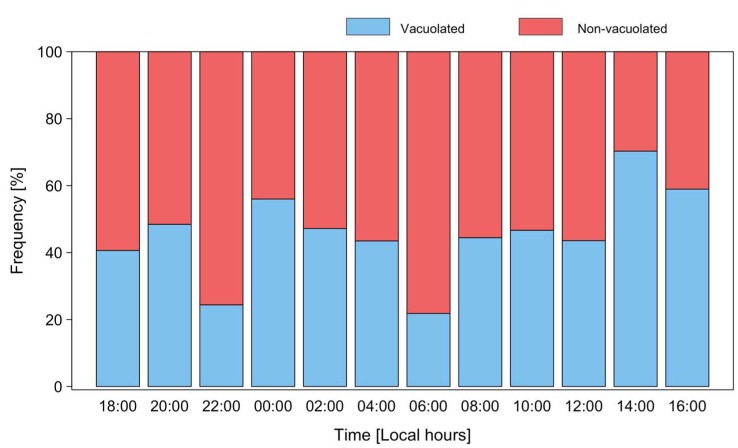
Distribution of frequencies of vacuolated (blue) and non-vacuolated (red bars) cells of *P. rotundatum* during a 24 h cycle from 16 to 17 January 2018.

**Figure 7:**
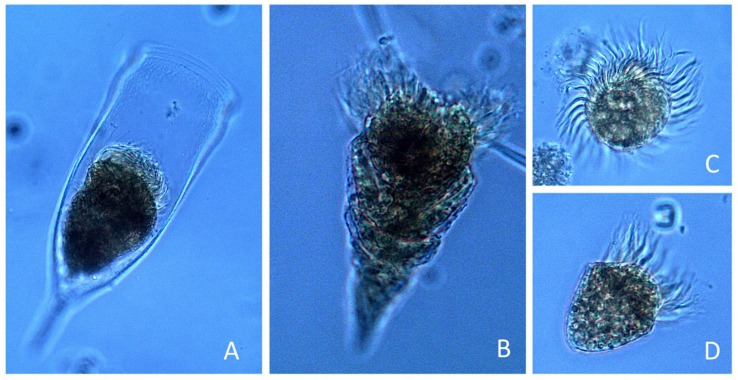
Micrographs of ciliates (genera) recorded in the net-haul samples: (**A**) *Favella*; (**B**) *Laboea*; (**C**) *Leegaardiella*; (**D**) *Strombidium*.

Micrographs of *P. rotundatum* cells taken throughout the cell cycle showed a high proportion of cells (up to 70%) full of digestive vacuoles. This observation provided evidence of recent heterotrophic feeding. The maximum frequency of vacuolated cells was observed on 17 January at 14:00, but a clear trend indicating a circadian rhythm in feeding behavior was not observed (Figure 6). Different species of co-occurring microplanktonic ciliates belonging to the genera *Favella*, *Laboea*, *Leegaardiella*, and *Strombidium* were observed (Figure 7), but *T. fusus* was not detected. Unfortunately, *Phalacroma* cells were not seen attached to ciliates in the feeding process to provide hints of the selected prey species.

## 4. Discussion

The heterotrophic dinoflagellate *P. rotundatum* usually occurs in low cell densities, rarely exceeding a few hundred cells per liter, and there is hardly any information on the environmental conditions associated with the proliferation of this species. This study resulted from an ad hoc sampling prompted by the observation of high numbers of this species in vertical net-haul samples during a routine visit to an oceanographic station in Reloncaví Sound. Net-haul samples allowed us to describe, for the first time, the cell cycle and to estimate in situ division rates and feeding status (well-fed vacuolated cells) of *P. rotundatum.*

### 4.1. Potential Multispecific Trophic Relationships in P. rotundatum

Hansen [7] described the feeding mechanism, known as myzocytosis, that *P. rotundatum* employs to prey on *T. fusus*, a ciliate 2–3 times larger than itself, and presented the specific predation on a ciliate by a heterotrophic dinoflagellate as a new trophic link in marine planktonic food webs. The dinoflagellate pierced the lorica of the ciliate with a feeding peduncle and suctioned its content into digestive vacuoles. After feeding, the *Phalacroma* cells appeared swollen, with a more rounded shape and a larger volume. A very specific predator–prey relationship was described, as only *T. fusus* was seen to be preyed upon by *Phalacroma* in field-sample incubations from a fixed station in southern Kattegat, Denmark, between August and December 1991. Hansen [7] was able to grow *P. rotundatum* in the laboratory with *T. fusus* fed on *Heterocapsa triquetra*.

In the present study, the high proportion of vacuolated cells of *P. rotundatum* was the unambiguous sign of recent phagotrophic feeding upon a good density of the adequate prey. Different microplanktonic ciliates belonging to the genera *Favella*, *Laboea*, *Leegaardiella*, and *Strombidium* co-occurred with *Phalacroma*, but not a single specimen of *Tiarina* was found throughout the whole 24 h intensive sampling. It must be concluded that ciliate species other than *T. fusus* served as prey of *Phalacroma* in the Chilean Fjord. This result differs from that of Hansen [7], who identified a single prey, *T. fusus*, for *P. rotundatum* in the southern Kattegat Sea. Molecular studies are ongoing to determine if DNA sequences in the dinoflagellate plastids are compatible with DNA plastid sequences from co-occurring microplanktonic ciliates.

### 4.2. Environmental Conditions during P. rotundatum Proliferation in Reloncaví Sound and In Situ Growth

Environmental conditions associated with the proliferation of *P. rotundatum* in January 2018 were very similar to those observed during the same period in the preceding (2017) and the following year (data not shown). These conditions are characterized by a strong seasonality in vertical water column structure, with maximal stratification and Brunt–Väisälä frequency estimates in spring–summer and weaker values in the autumn. Unfortunately, only vertical net hauls were collected, so it was not possible to relate *Phalacroma* densities with fine-scale water column structure. Likewise, it was not possible to describe relationships between the vertical distribution of *Phalacroma* and that of potential ciliate prey, nor to compare cell densities with those from similar situations in previous years because they were not counted.

The 24 h cycle study showed that *P. rotundatum* has a very synchronized phased cell division and a high (nearly 0.5 d^−1^) division rate of almost one doubling per day, a normal response observed in mixotrophic dinoflagellates after prey feeding. A division rate “*µ*” of 0.69 d^−1^, i.e., one doubling per day, is the highest observed within *Dinophysis* species in the Galician Rías using the same approach, but values between 0.2 and 0.3 d^−1^ are the most frequent [10,11]. During a cell cycle study of a dense population of *Dinophysis norvegica* in the Baltic Sea, Carpenter et al. [26] did not observe a diel rhythm of DNA synthesis and suggested that *D. norvegica* was either not dividing synchronously (they considered asynchronous division was common in heterotrophs) or not dividing at all. Digestive vacuoles were lacking. Similar observations were reported by Gisselson et al. [27] in a *Dinophysis acuminata* population in the Gullmar Fjord, Skagerrak, Sweden. These authors suggested that heterotrophic growth in the dark (in mixotrophic dinoflagellates) may reduce the degree of diurnal synchronization of the cell cycle or even allow asynchronous division during all stages of the diel cycle. Results from previous cell cycle studies with *Dinophysis* species [10,11,28] and the present study of *Phalacroma* differ from these views and suggest that those authors were dealing with populations that were not dividing at all. In the present study, synchronized division coincided with a high proportion of vacuolated cells and high division rates (0.46 d^−1^) in a colorless heterotrophic dinoflagellate, *Phalacroma rotundatum.* Our results agree with observations by Skovgaard [29] and Rodríguez et al. [30] on facultative mixotrophic species of the genus *Fragilidium*. These species can grow as autotrophs, but when exposed to their prey and the opportunity to switch to heterotrophic feeding, division rates were much higher.

### 4.3. Implications for HAB Monitoring and DSP Toxicity

The controversy about the toxicity of *P. rotundatum* will not be solved until different strains of *P. rotundatum* are established in culture and their potential toxicity tested. Miles et al. [31] and González-Gil et al. [19] found toxins in picked cells of *P. rotundatum* cells only when they were accompanying toxic species of *Dinophysis* (*D. acuminata, Dinophysis acuta, D. norvegica*). González-Gil et al. [19] suggested that *P. rotundatum* does not produce toxins but may take them up from *Dinophysis* recently preyed upon by ciliates and thus act as a vector of DSP toxins. In these circumstances, *P. rotundatum* would pose a risk to shellfish contamination and the aquaculture industry [32]. This possibility needs to be evaluated when its blooms co-occur with toxin-producing species of *Dinophysis*. Nevertheless, it should be borne in mind that each vector species has a specific response and requires different times to metabolize each kind of toxin taken up [33].

During the cell cycle study in Reloncaví Sound, the microplankton community was dominated by diatoms, there were isolated cells of *D. acuminata,* and *P. rotundatum* was the dominant dinoflagellate. The official monitoring of lipophilic toxins carried out by the Chilean Fisheries Development Institute (IFOP) showed positive values in ribbed mussels (“cholga”) *Aulacomya atra* in January 2018 in Isla Caicura (41°43′59′’S; 72°40′59′’W), a station in Reloncaví Sound very close to the buoy station. DSP toxins were also present in shellfish from the six sampling stations from Reloncaví Fjord. Considering the few *D. acuminata* cells observed during this study, the possibility that *P. rotundatum* recently fed *Dinophysis*-containing tintinnids acted as a vector of DSP toxins to shellfish filter-feeders cannot be discounted. Ongoing lipophilic shellfish toxin analyses of the net-haul samples by liquid chromatography coupled to tandem mass spectrometry (LC–MS/MS) will confirm or discard this possibility.

## 5. Conclusions

A cell cycle study of a population of the heterotrophic dinoflagellate *P. rotundatum* showed patterns similar to those of mixotrophic *Dinophysis* species. A highly synchronized phased cell division was observed, with a peak 3 h after sunrise, during strong summer stratification in a Patagonian Fjord in southern Chile. High division rates (0.46 d^−1^) may have been triggered by a high prevalence (>70% at 14:00) of well-fed (vacuolated) cells, which did not show a clear circadian rhythm in their feeding behavior. The absence of *Tiarina fusus*, considered the specific prey for *Phalacroma* in Northern Europe, suggests feeding on other yet to be identified ciliate prey.
